# Comparative analysis on lung transcriptome of *Mycoplasma ovipneumoniae* (Mo) - infected Bashbay sheep and argali hybrid sheep

**DOI:** 10.1186/s12917-021-03040-3

**Published:** 2021-10-13

**Authors:** Zengqiang Li, Zhihui Du, Jie Li, Yanming Sun

**Affiliations:** College of Animal Science and Technology, Shihezi University, Shihezi, Xinjiang, 832003 China

**Keywords:** Bashbay sheep, Argali hybrid sheep, *Mycoplasma ovipneumoniae*, Comparative transcriptome analysis

## Abstract

**Background:**

Bashbay sheep (Bbs) has a certain degree of resistance to *Mycoplasma ovipneumoniae* (Mo), however, Argali hybrid sheep (Ahs) is susceptible to Mo. To understand the molecular mechanisms underlying the difference of the susceptibility for Mo infection, RNA-sequencing technology was used to compare the transcriptomic response of the lung tissue of Mo-infected Bbs and Ahs.

**Results:**

Six Bbs and six Ahs were divided into experimental group and control group respectively, all of them were experimentally infected with Mo by intratracheal injection. For collecting lung tissue samples, three Bbs and three Ahs were sacrificed on day 4 post-infection, and the others were sacrificed on day 14 post-infection. Total RNA extracted from lung tissue were used for transcriptome analyses based on high-throughput sequencing technique and bioinformatics. The results showed that 212 (146 up-regulated, 66 down-regulated) DEGs were found when comparing transcriptomic data of Bbs and Ahs at 4th dpi, besides, 311 (158 up-regulated, 153 down-regulated) DEGs were found at 14th dpi. After GO analysis, three main GO items protein glycosylation, immune response and positive regulation of gene expression were found related to Mo infection. In addition, there were 20 DEGs enriched in these above items, such as SPLUC1 (BPIFA1), P2X7R, DQA, HO-1 and SP-A (SFTPA-1).

**Conclusions:**

These selected 20 DEGs associated with Mo infection laid the foundation for further study on the underlying molecular mechanism involved in high level of resistance to Mo expressed by Bbs, meanwhile, provided deeper understandings about the development of pathogenicity and host-pathogen interactions.

**Supplementary Information:**

The online version contains supplementary material available at 10.1186/s12917-021-03040-3.

## Background

Bashbay sheep (Bbs), one of the finest local sheep breeds in Xinjiang of China, possesses some excellent characteristics, such as fast growth and development, high meat production, and resistance to disease [1]. Wild argali sheep, a second-grade animal under state protection in China, is one of the biggest wild sheep with some merit including stress tolerance, rapid growth and donkey-like body shape without too much fat [2]. However, argali sheep were vulnerable to Mycoplasma ovipneumonia, a respiratory disease caused by *Mycoplasma ovipneumoniae* (Mo) infection [3]. After hybridizing adult male Argali sheep with adult female Bbs, a new improved variety known as Argali hybrid sheep (Ahs) was reported to be raised for the past few years [4]. By the further study on the adaptive analysis of the above cross lambs (Ahs), Helatti et al. has firstly discovered that their large donkey-like physique were inherited from paternal Argali sheep with stable hereditary properyies, meanwhile, they inherited the traits of rapid growth and good palatability from paternal Bbs without exception [5].

Many diseases of sheep and goats caused by mycoplasma infections are always considered to be responsible for the economic costs of sheep husbandry around the world, due to the falling of milk yield, high morbidity rate and death rate [6]. Mo infection is regarded as the specific etiology of Mycoplasma ovipneumonia known as one of the most common chronic respiratory diseases of sheep, and a long-term coughing syndrome, sneeze and progressive weight loss always characterizes this disease [7]. Moreover, an endemic disease of sheep always present in parts of New Zealand about 20 years ago was defined as chronic non-progressive pneumonia, and Mo was thought to be associated with the occurrence of the above pneumonia pandemic at that time [8]. In recent years, the fact that there are some differences between Bbs and Ahs in response to Mo infection has been proved, which is that Bbs have obvious resistance to Mo infection, but to which Ahs was extremely susceptible [9].

In spite of the completion of extensive research on the pathogenic mechanism of Mo in the past, the undeniable fact is that we know little about the precise molecular mechanisms related to both the host’s resistance and susceptibility to this pathogen at present. To find the molecular basis of being resistant or susceptible to Mo, a comparative transcriptome analysis of lungs from Mo-infected Bbs and Ahs was carried out. In this study, Bbs and Ahs were chosen to conduct artificial infection experiments, their lungs were collected at 4th dpi and 14th dpi respectively for further experiment including high-throughput sequencing, identification of differentially expressed genes (DEGs) and Gene Ontology (GO) enrichment analysis of selected DEGs related to Mo infection. To some extent, DEGs screened in this study may provide more clues as to the molecular mechanism related to Mo infection, laying foundation for further study on the relationships between host and pathogenic microorganisms.

## Results

Serum from both experimental BbS and Ahs were tested negative for Mo infection by Mo-Ab kit at 4th dpi but positive at 14th dpi. Nasal swabs samples were collected at 4th dpi and 14th dpi respectively, and then they were cultured under suitable conditions (37°C, 5% CO_2_) for the identification of Mo. These red nutrient solutions turned yellow at 5th day after the inoculation of samples isolated from nasal cavity of experimental lambs. In addition, both Giemsa staining and biochemical identification were applied to verify the existence of Mo in the yellow medium. Observation of the sample stained with Giemsa microscopically proved a fact that there were so many spherical and pleomorphic mycoplasmas existed exactly. The results of biochemical identification showed that glucose fermentation test, digitalis sensitivity test, tetrazolium chloride reduction test, hemadsorption test and hemolytic test (β-hemolysis) were all positive, but arginine hydrolysis test was negative, indicating that the observed mycoplasma species was no other than Mo. These evidences confirmed that the model of Mo-induced mycoplasma ovipneumonia was built successfully. Moreover, after lambs were challenged with Mo strain, the clinical symptoms of pneumonia: inappetence, cough, sternutation and runny nose continued to appear. One notable feature of the disease was that the stage of fervescence is short. Rectal temperature of these lambs went up (40–41.5 °C) temporarily at 2nd and 3th dpi, and then returned to normal (38–39.5 °C) after 2 days approximately.

**Fig. 1:**
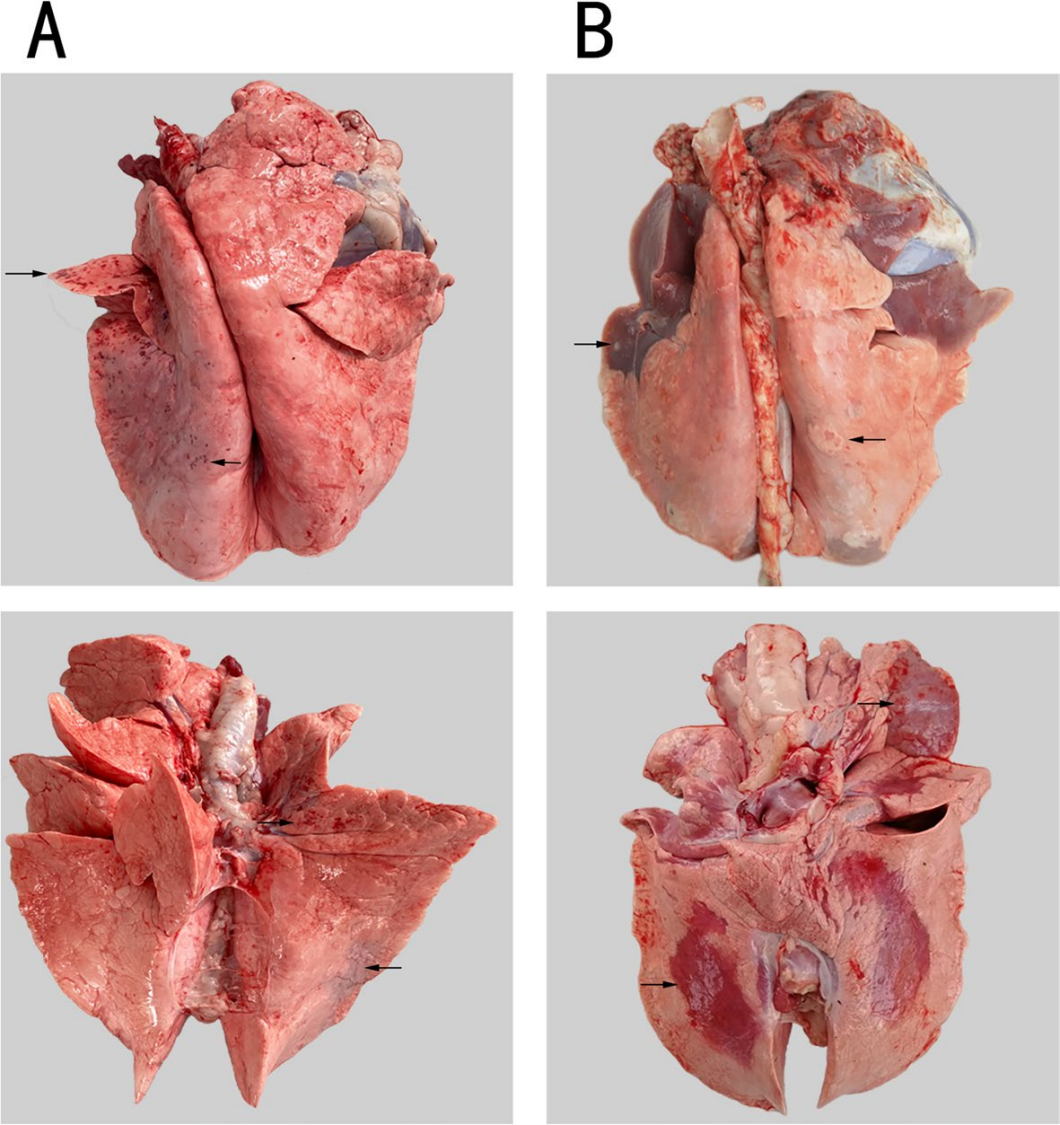
Gross lesions of lungs from Bbs and Ahs at 14th dpi. **A** The lungs isolated from the thoracic cavity of Bashbay sheep, only a small amount of hemorrhagic spots were observed on the face of lungs; **B** The lungs isolated from the thoracic cavity of Argali hybrid sheep, there were much severer inflammatory lesions in lungs of Ahs than Bbs, including large-scale red and gray hepatization and bulge nodules. Typical lesions are pointed out by the black arrows in the above figures

After being infected with Mo, the differences of clinical symptoms and pulmonary lesions of the experimental lambs in four groups were observed clearly. The lambs in both Z-4-dpi group and Z-14-dpi group experienced the most common clinical symptoms of Mo infections including coughing, dyspnea, fever, sneezing and prostration. Meanwhile, two Ahs in Z-14-dpi group also suffered from diarrhea. However, the lambs in B-4-dpi group and B-14-dpi group experienced it in a relatively mild form called subclinical infection. The anatomic lesions of the lung from Bbs and Ahs were observed respectively as shown in Fig. 1. Macroscopic pathological changes of the lungs of Bbs were only slight, a small amount of hemorrhagic spots were observed on the face of lungs (Fig. 1A). By contrast, there were much severer inflammatory lesions in lungs of Ahs than Bbs, including large-scale red and gray hepatization and bulge nodules (Fig. 1B). The contrast between the clinical symptoms and pulmonary lesions of Bbs and its counterpart of Ahs reinforced the past findings that Bbs has a certain resistance to Mo; on the contrary Ahs were susceptible to Mo infection. In addition, the observed pulmonary lesions of these two kind of sheep demonstrate our success in artificial infection of Mo, which established the foundation for the next experiment in RNA-seq of lungs.

There were twelve RNA samples extracted from the lung tissues of the lambs in four group, and the results of quality detection showed that all of them reached the sequencing standard. We got more than 5.2 × 10^7^ raw sequence reads with each sample after Illumina high-throughput second generation sequencing, and then low-quality data was screened out from original data. After the quality control of reads, the clean reads number of each sample were over 5.0 × 10^7^.

It is of utter importance to secure high quality sequencing data, enabling the identification of the difference in gene expression between Bbs and Ahs in response to experimental Mo infection. Any sample’s proportion of valid bases was found greater or equal to 95.74%. We found that all samples’ Q30 values meet the basic criterion (Q30 ≥ 90%) from the result of quality control (95.60–97.56%). All the obtained sequencing data were considered to be fairly reliable, because their error rates were kept in a reasonable range. In addition, the comparison between GC content of sequencing results (48.50–51.50%) and GC content (46.88%) in exons of ovine genome reported on NCBI website proved the availability of these data we got, indicating that they are suitable for subsequent analysis.

In this study, we mapped the selected high-quality clean reads to the genome (*Ovis aries*) of sheep using TopHat v2.0.12. There were 83.35–86.46% of total cleaning reads totally mapped to sheep genome and 74.19–78.90% of which were uniquely mapped to a certain region within sheep genome. The results showed both the percentage of totally mapped reads and uniquely mapped reads satisfied a special criterion that the rate of mapped rates must be more than 70%. In addition, any percentage of multi-mapped reads in each sample (8.28% ± 0.60%) was less than 10%, which is up to standard. In conclusion, any result of the alignments achieved their own relevant quality requirement, indicating that these data were practicable for subsequent analysis on DEGs.

The DEGs related to Mo infection in lungs of Bbs and Ahs were picked out. We identified 212 DEGs when comparing B-4-dpi group with Z-4-dpi group (4d-B/Z group), among which, there were 146 up-regulated genes (Table S1) and 66 down-regulated genes (Table S2), accounting for 68.87 and 31.13% respectively. In addition, we discovered 311 DEGs by comparing Bbs-14-dpi group with Ahs-14-dpi group (14d-B/Z group). In which, 158 genes upregulated (Table S3) and the rest 153 gene downregulated (Table S4), the former accounted for 50.80%, and the latter accounted for 48.20%.

**Fig. 2:**
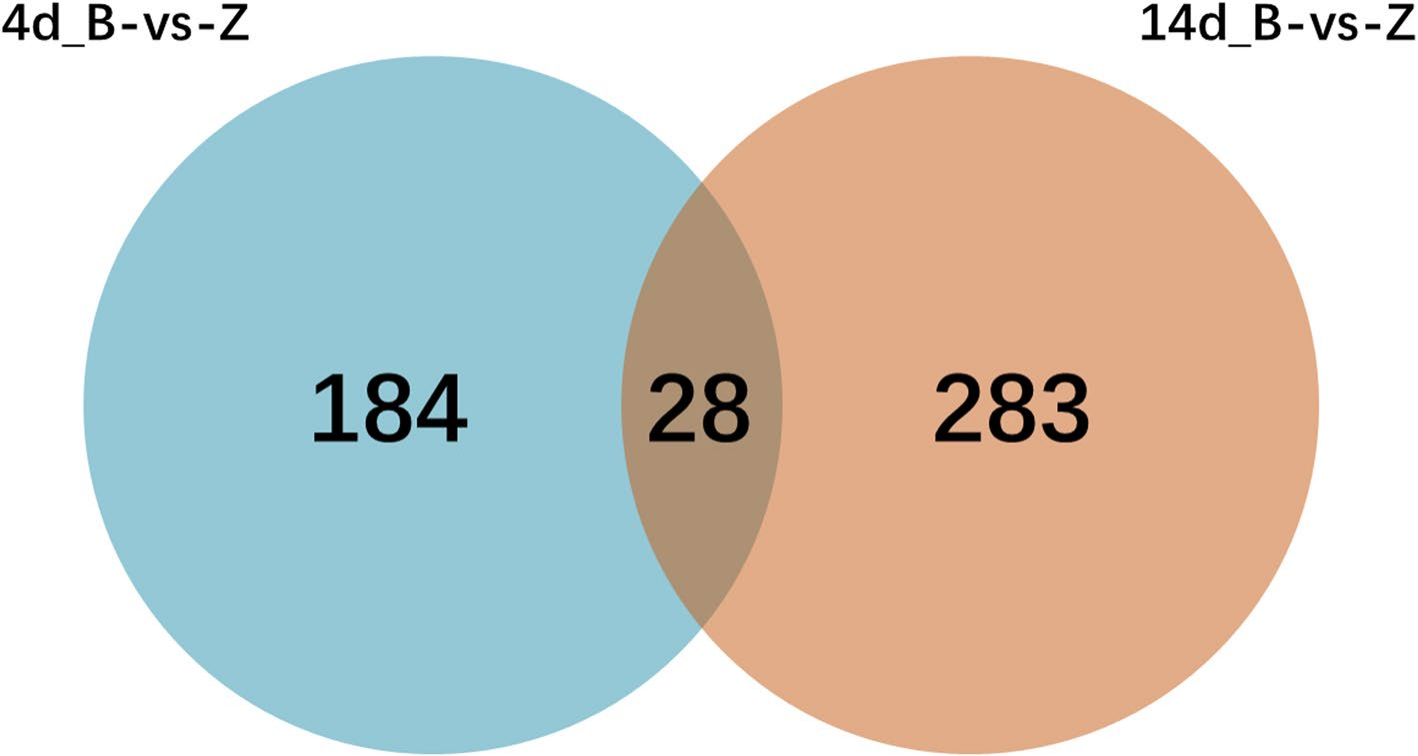
Wayne chart showing the number of genes with significant differences in relative abundance induced by duration of infection in two sheep breeds when comparing Bbs with Ahs at a false discovery rate (FDR) cutoff < 0.05. 184 DEGs were identified in the 4d-B/Z group between Bbs and Ahs; 283 DEGs were identified in the 14d-B/Z group between Bbs and Ahs; 28 DEGs were identified in both 4d-B/Z group and 14d-B/Z group

Analysis on the relations between 4d-B/Z group and 14d-B/Z group were conducted through the Wayne chart way (Fig. 2). As shown in the figure, 4d-B/Z group had 184 exclusive DEGs, and there were 283 DEGs which were proper to 14d-B/Z group. In addition, 28 common DEGs existed in both groups.

**Fig. 3:**
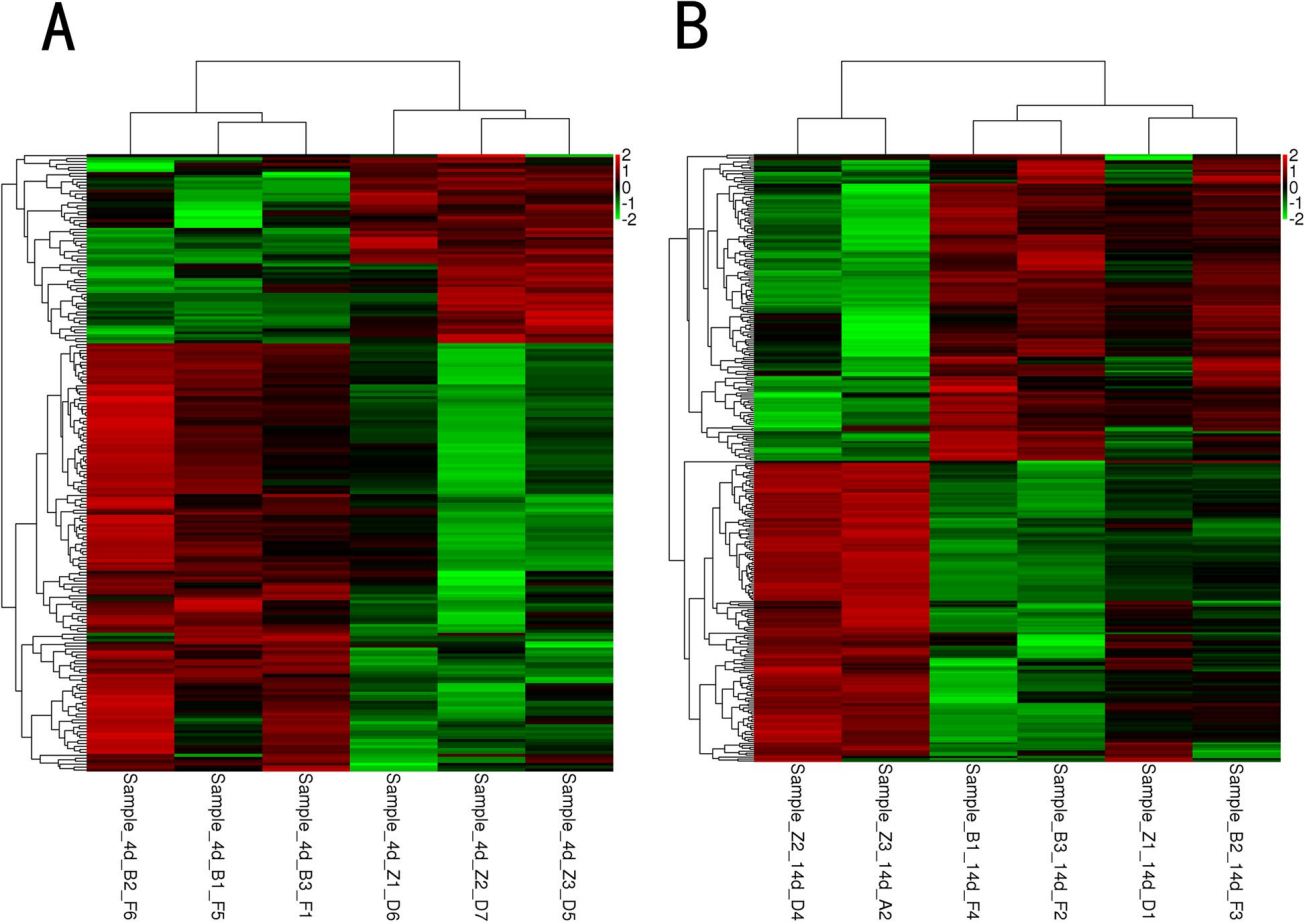
The result of hierarchical clustering of identified DEGs between different groups. **A** The hierarchical clustering of 4th-dpi group including Bbs and Ahs; **B** hierarchical clustering of 14th-dpi group including Bbs and Ahs. In this heat maps, one small square represents one gene, and its color represents the expression level of the gene; each row represents the expression level of each gene in different samples, and each column represents the expression level of all genes in each sample

Heat maps were made according the FPKM values to display the results of clustering analysis more intuitively, in which one small square represents one gene and its color represents the expression level of the gene. Each row represents the expression level of each gene in different samples, and each column represents the expression level of all genes in each sample. As can be seen from Fig. 3A, all samples in B-4-dpi group were clustered together; all samples in Z-4-dpi group were clustered together. Similarly, all samples in B-14-dpi group were clustered together. However, the results in Z-14-dpi group were somewhat different: two samples in which were clustered together and the rest one was clustered together with B-14-dpi group (Fig. 3B). The above results indicated that Bbs and Ahs had their own gene expression pattern and the gene expression pattern of Ahs changed at 14th dpi.

In order to identify GO terms in which the DEGs enriched largely, GOseq R package were used for GO enrichment analysis on the DEGs related to Mo infection. In our study, a total of 758 GO terms shared by the Bbs and Ahs in 14th-dpi group in response to the Mo infection were identified, including 464 biological process terms (61.22%), 205 molecular function terms (27.04%), and 89 cellular component terms (11.74%). In addition, 784 GO terms were identified in 14d-B/Z group embracing 484 biological process terms (61.73%), 194 molecular function terms (24.75%), and 106 cellular component terms (13.52%).

**Fig. 4:**
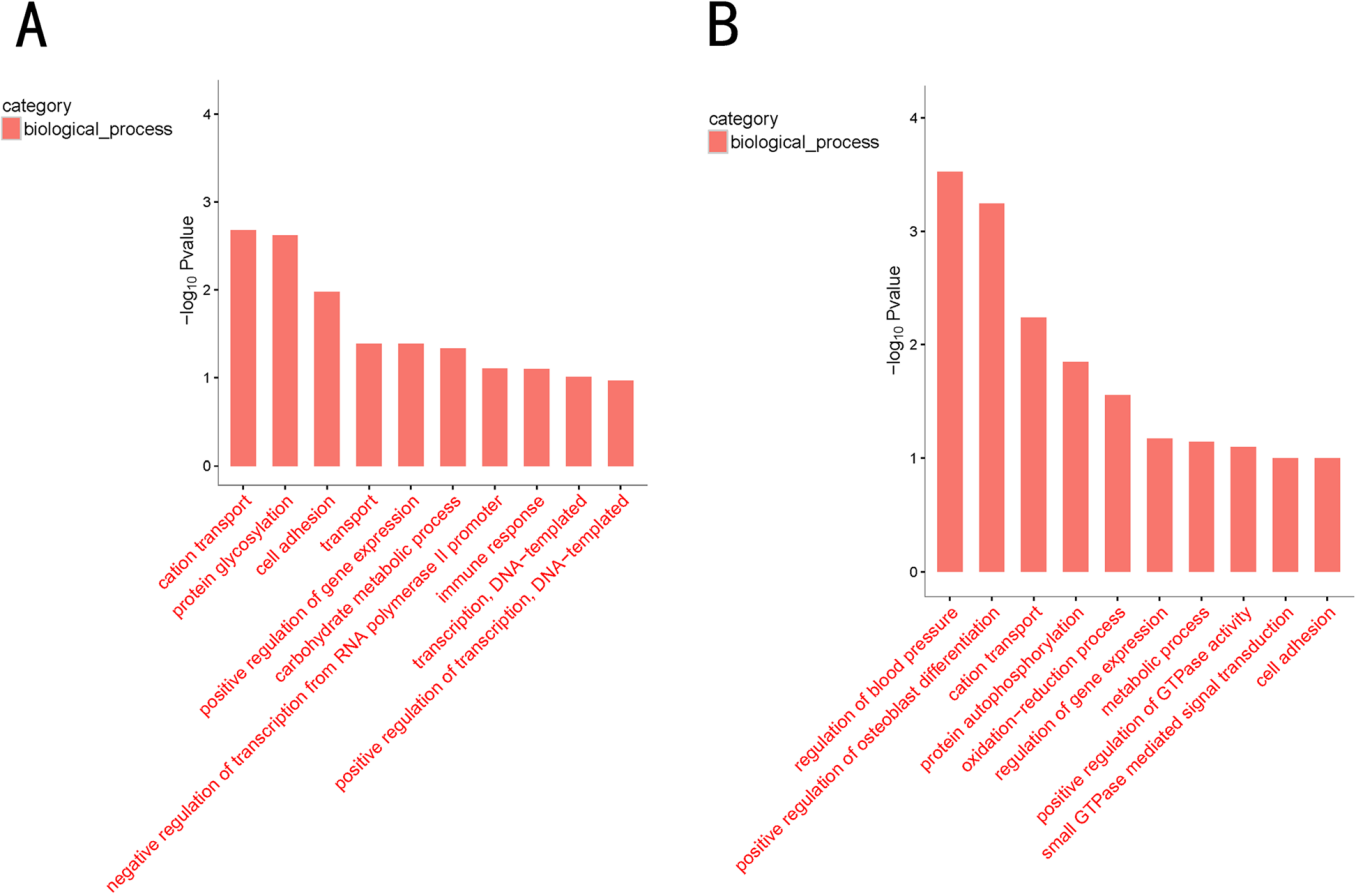
The top 10 GO terms in biological process of experimental sheep infected with Mo. **A** The top 10 GO terms of 4d-B/Z group; **B** The top 10 GO terms of 14d-B/Z group. The Go items related to biological process with more than 2 genes were screened, and then they were sorted according to the corresponding -log10*P*value. In the figure, the abscissa is the name of Go items, and the vertical axis is -log10*P*value

Moreover, as is shown in Fig. 4A, the top 10 GO terms for biological processes in 4th-dpi group included “cation transport,” “protein glycosylation,” “cell adhesion,” and “transport” et al. Likewise, the top 10 GO terms for biological processes in 14th-dpi group can be seen clearly from Fig. 4B, including “regulation of blood pressure,” “positive regulation of osteoblast differentiation,” and “cation transport” et al.

Functional annotations and classifications of the GO terms in which DEGs enriched were conducted to determinate which physiological functions were influenced by Mo infection. Both the results of 4d-B/Z group and 14d-B/Z group were shown as Table 1, including protein glycosylation (*p* = 2.41E-03), immune response (*p* = 7.94E-02), positive regulation of gene expression (*p* = 4.13E-02) et al.

**Table 1:** The most representative DEGs-enriched GO items related to Mo infection

Comparison	GO ID	GO Term	P-value	DEGs number
4d-B/Z	GO:0006486	protein glycosylation	2.41E-03	3
	GO:0007155	cell adhesion	1.06E-02	5
	GO:0010628	positive regulation of gene expression	4.13E-02	4
	GO:0006955	immune response	7.94E-02	3
14d-B/Z	GO:0046777	protein autophosphorylation	1.43E-02	4
	GO:0055114	oxidation-reduction process	2.79E-02	11
	GO:0010468	regulation of gene expression	6.74E-02	3
	GO:0008152	metabolic process	7.21E-02	17
	GO:0007264	small GTPase mediated signal transduction	1.00E-01	4
	GO:0007155	cell adhesion	1.00E-01	4

A total of 20 DEGs involved in Mo infection were screen out by conducting comprehensive analysis on the results of sequence alignment and GO enrichment analysis, such as SPLUC1, P2X7R, DQA, SOCS3, HO-1 and SP-A et al., details are shown in Table 2.

**Table 2:** Data concerning differentially expressed genes related to Mo infection

Gene symbol	Description	Fold-change		*p*-value	
		4/0d	14/0d	4/0d	14/0d
MYD88	myeloid differentiation primary response 88	2.08	0.86	0.01	0.02
NFKB1	nuclear factor kappa B subunit 1	2.07	0.86	0.001	0.001
IL1B	interleukin 1 beta	3.03	1.25	0.43	0.006
IL10	interleukin 10	2.06	0.66	0.05	0.018
TLR2	toll-like receptor 2	0.87	4.81	0.007	0.017
TLR6	toll-like receptor 6	1.68	2.75	0.01	0.013
SP-A (STFPA1)	surfactant protein A1	0.50	0.55	0.021	0.019
P2X7R	purinergic receptor P2X, ligand gated ion channel, 7	2.05	0.45	0.0014	0.0032
DQA	HLA class II histocompatibility antigen, DQ alpha 2 chain	4.47	0.71	0.021	0.0043
SOCS3	suppressor of cytokine signaling 3	4.79	0.34	0.031	0.058
HO-1 (HSP32)	hemeoxygenase 1	0.68	5.79	0.0032	0.0015
NFE2	nuclear factor, erythroid 2	0.59	3.38	0.012	0.041
SOD2	superoxide dismutase 2	2.48	0.40	0.0065	0.033
GATA3	GATA binding protein 3	3.17	0.55	0.046	0.026
ISG15	ISG15 ubiquitin-like modifier	0.44	4.67	0.037	0.025
ISG20	interferon stimulated exonuclease gene 20 kDa	0.47	4.61	0.041	0.0010
NQO1	NAD(P)H dehydrogenase, quinone 1	0.87	3.23	0.0083	0.0056
IRF5	interferon regulatory factor 5	0.62	3.03	0.042	0.016
MAP2K6 (MKK6)	mitogen-activated protein kinase kinase 6	0.54	3.47	0.0055	0.023
BPIFA1 (SPLUNC1)	BPI fold containing family A member 1	4.71	9.72	0.056	0.0025

**Fig. 5:**
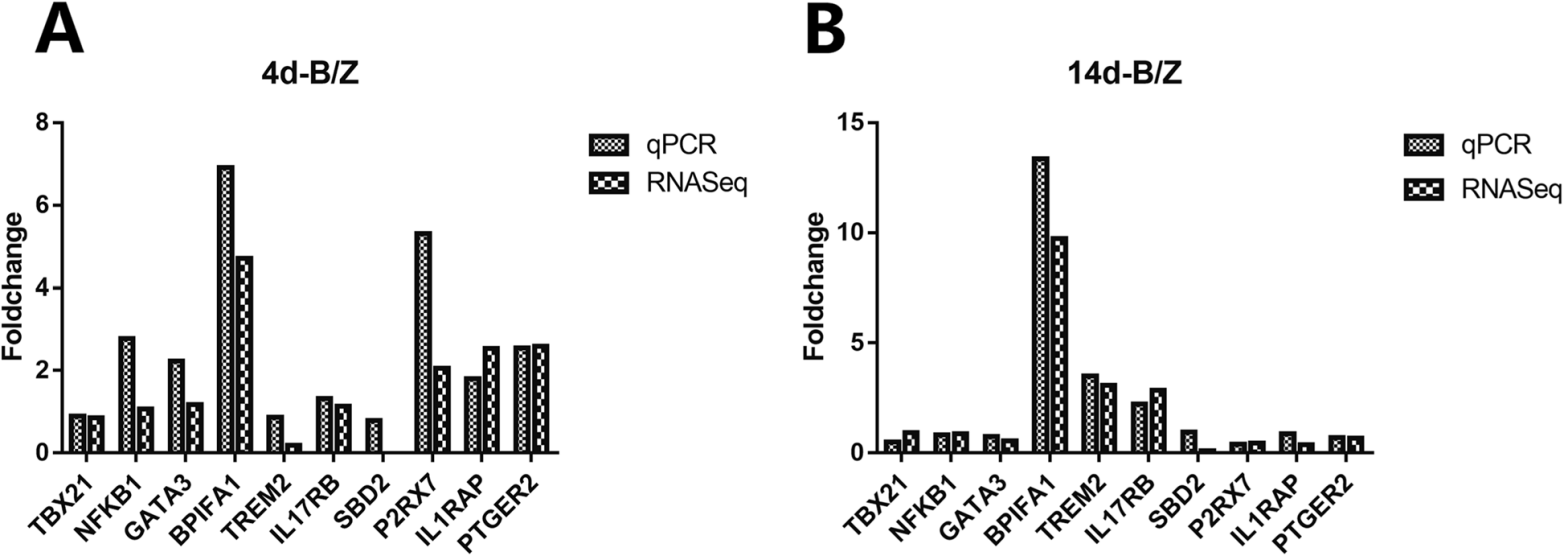
The verification of RNA-Seq results by fluorescence quantitative PCR. Relative expression levels calculated from standard curves were normalized to the endogenous control GAPDH gene. TBX21: T-box transcription factor 21; NFKB1: nuclear factor kappa B subunit 1; GATA3: GATA binding protein 3; BPIFA1: BPI fold containing family A member 1; TREM2: triggering receptor expressed on myeloid cells 2; IL17RB: interleukin 17 receptor B; SBD2: beta defensin 2; P2RX7: purinergic receptor P2X 7; IL1RAP: interleukin 1 receptor accessory protein; PTGER2: prostaglandin E receptor 2

The RNA-seq results of randomly selected genes (ten DEGs and GAPDH as reference gene) were validated according to the same sample set by real-time (q) RT-PCR. The expression results of TBX21, NFKB1, GATA3, BPIFA1, TREM2, IL17RB, SBD2, P2RX7, IL1RAP, PTGER2 obtained using qRT-PCR were in good agreement with the RNA-seq results (Fig. 5).

## Discussion

It’s well known that Mycoplasma ovipneumonia, a major health issue for sheep industry worldwide, causes significant losses to farmers in declining production. There exist significant differences in extent of disease caused by Mo infection between Bbs and Ahs, which was proved in the early clinical research completed by our team [9]. During the above experiment, a notable feature of these two kinds of sheep respectively was discovered: Bbs has some resistance to Mo, Ahs was susceptible to Mo infection on the contrary. Carson et al. [10] reported that Agali sheep suffered severe respiratory disease when they were kept with domestic sheep experimentally infected with Mo, while domestic sheep experienced no respiratory symptom from beginning to end, indicating Agali sheep was very vulnerable to this pathogen. We believe that the susceptibility to Mo of Ahs was inherited from its parental Agali sheep based on the enlightenment of the above research. Bbs and Ahs were chosen as ideal experimental animals in this study to unravel the molecular mechanisms related to the immune resistance and susceptibility of sheep to Mo. After the analysis upon pulmonary transcriptome of these two kind of sheep were performed by RNA-seq technique, 20 DEGs closely associated with Mo infection were identified, such as SPLUC1, P2X7R, DQA, SOCS3, HO-1 and SP-A.

SPLUNC1 (BPIFA1) was transcribed by the genes of PLUNC gene family. It is a member of the BPI/LBP (bactericidal/permeability-increasing protein/lipopolysaccharide-binding protein) family which can bind to lipopolysaccharide (LPS) with high-affinity [11]. SPLUNC1-deficient (SPLUNC1−/−) mice generated by gene knockout technology and hSPLUNC1 transgenic mice were infected with *Mycoplasma Pneumoniae* (Mp) experimentally to explore the in vivo functions of SPLUNC1 by Gally et al., [12] finding that pulmonary Mp load of SPLUNC1^−/−^ mice was three times as much as its counterpart of wild type controls, and the pulmonary Mp load of wild-type mice was three times over hSPLUNC1 transgenic mice. The above mice experiment may reinforce a notion that SPLUNC1 functions as an immune molecule under the situation of Mp infection, which may help to resist infection [13]. In this study, the content of SPLUNC1 mRNA in lungs of Bbs was 4.71 times higher than that of Ahs in 4th-dpi group and the content of SPLUNC1 mRNA in lungs of Bbs was 9.72 times higher than that of Ahs in 14th-dpi group, which indicates that the resistance that Bbs possesses to Mo infection may be associated closely with the high expression of SPLUNC1 gene.

Excessive immune response induced by toxic materials and microorganisms is one of the main reasons for lung injury, [14] resulting in the local inflammatory response. The interaction of various cells (immune, endothelial, and parenchymal cells) and the expression of several bioactive molecules (cytokines, chemokines, and adhesion molecules) are closely associated with the development of inflammation [15]. Purinergic ligand-gated ion channel 7 receptor (P2X7R), a plasma membrane receptor for extracellular ATP, functions as a signaling molecule, and assorted adverse external factors act as the stimulus to the production of them. Accruing evidence suggests that P2X7R is involved in the signaling pathway in inflammation [16].. Moncao-Ribeiro et al. have attempted to explore the role that P2X7R plays in silica-induced lung pathological changes by erecting a silica-induced pulmonary fibrosis model of mice, confirming that P2X7R is capable of modulating lung inflammatory, fibrotic, and functional changes as a regulator [17]. The pulmonary inflammation and fibrosis of P2X7R gene knockout mice in his study was less severe than that of the mice in control group, indicating the expression of P2X7R gene was closely associated with the pathogenesis and exacerbation of lung injury. Our data of DEGs showed that the expression level of P2X7R mRNA in lungs of Ahs was twice as much as that of Bbs in 14th-dpi group, which may be one of the reasons why the clinical symptoms and lung injury of Mo-infected Ahs were more severe than Bbs.

DQA gene, a member of the major histocompatibility complex (MHC) gene family in vertebrates, is involved in the expressions of many transmembrane proteins [18]. The expression product of DQA gene can present peptides to specific receptors on the surface of T cells in the immune system, which is one of the triggers for various immune responses [19]. It was found that Mo can not only inhibit the production of complement and the expression of IgG receptor on the surface of macrophage, but also provoke an overexpression of MHC class II molecules on the surface of macrophage [20]. The overexpression of MHC class II molecules has previously been found to be responsible for immune dysfunction, resulting in immunological disease ultimately [21]. In the present study, the pulmonary DQA mRNA expression level of Ahs was five times as higher as that of Bbs. The DQA gene overexpression of Ahs would result in the excess of MHC class II molecules, which may account for the observed phenomenon at 14th dpi that the lung injury of Mo-infected Ahs was more severe than that of Mo-infected Bbs.

Suppressor of cytokine signaling (SOCS) proteins are proved to be feedback inhibitors of the Janus kinase/signal transducer and activator of transcription (JAK/STAT) signaling pathway [22]. Up-regulated SOCS3 proteins induced by the stimulation of several signals in macrophages contribute to the inhibition of the expression and release of inflammatory factors so as to alleviate inflammatory reaction [23]. In addition, Hu et al. has confirmed that SOCS3 proteins considered as a regulator of various signaling pathways could inhibit the expression of NO, TNF-α, IL-6 and GM-CSF in inflammatory response, consequently reducing the injury due to over reaction of inflammation [24]. Higher expression of SOCS3 gene (about 4.9 times) was observed in lungs of Bbs than that of Ahs at 4th-dpi in this study, which may be a major reason why both of the pulmonary lesions and clinical symptoms of Bbs were all slighter than that of Ahs.

Haem-oxygenase-1 (HO-1), also known as Heat Shock Protein (HSP32), is the most researched isoenzyme in heme oxygenase which is an important antioxidize system acting essential biology functions internally. Not only does HO-1 act as catalysts to the reaction during heme degradation, but also HO-1 has also been previously known to protect the body against the damaging effects of free radicals and acute inflammation in the progress of some diseases [25]. A previous study in vitro indicated that HO-1 expression was dramatically increased in THP-1 cells inoculated with lipid associated membrane proteins (LAMPs) of Mp, showing strong protective effect on THP-1 cells against Mp infection [26]. Similar studies on the biological function of HO-1 revealed the detailed mechanism to ameliorate inflammatory damage, which is accomplished by inhibiting the activation and decreasing the aggregation of reactive oxygen species (ROS) [27]. It should be noted that the expression level of HO-1 mRNA in the lungs of Bbs was much higher than that of Ahs (about 5.79 times) at 14th-dpi, thus, abundant HO-1 expressed in lungs of Mo-infected Bbs may play an important role in alleviating the injury of alveolar epithelial cell so as to protect lungs against virulence of Mo.

## Conclusions

We performed a comparative analysis upon transcriptome between Mo-infected Bbs and Ahs for the first time. In this study, a total number of 20 DEGs closely associated with Mo infection were identified using high-throughput RNA-seq technology and bioinformatics tools. What we found provides a theoretical basis for further study on the specific mechanism of the difference in resistance/susceptibility to Mo that comes from breed differences.

## Methods

### Animal grouping and artificial infection of Mo

Twelve disease-free sheep including six Bbs and six Ahs were kept within sheepfold in the experiment education demonstration center of animal hospital affiliated to Shihezi University. All of these experimental animals regardless of sex were approximately 2.5-month-old lambs and physically similar (15 ± 5 kg). Bbs were all purchased from a small and medium-sized sheep farm in Tacheng prefecture located in the northwest of Xinjiang; Ahs were obtained from a small breeding enterprise in 170th Regiment of the 9th Division, Xinjiang Production and Construction Corps, China. Before the proper start of experiment, 12 serum samples were isolated from venous blood of these lambs, and then they were utilized for detection of Mo infection by enzyme-linked immunosorbent assay (ELISA) using Mo-Ab ELISA kit bought at R&D Systems in Shanghai of China, to confirm that all the experimental lambs were not affected by Mo previously. Six Bbs were randomly divided into B-4-dpi group and B-14-dpi group, three per group; Six Ahs were randomly divided into Z-4-dpi group and Z-14 dpi group, three per group. The first two groups were treated as experimental group, and the remaining two groups were considered as control group. The study was approved by local institutional research ethics committee, and every step of experimentation on animals fully complied with relevant requirements to ensure their welfare.

Dr. Genqiang Yan, a distinguished professor in College of Animal Science and Technology under Shihezi University, was quite interested in our study. Hence, he gave Mo_1412_ strain to us for experimental infection, high similarity (up to 98%) between Mo 16S rRNA gene fragment and the counterpart from standard Mo Y-98 type strain. Artificial infection experiment of Bbs and Ahs by Mo was carried out according to the method Jiang et al. reported [28].

Mo-Ab ELISA kit (R&D Systems) was used for the detection of specific antibodies in sera against Mo at 14th dpi to determine whether these lambs were infected with Mo successfully. Meanwhile, for the purpose of identifying Mo, nasal swab samples of all lambs were collected and cultivated (37 °C, 5% CO_2_) for 5 days after being inoculated in mycoplasma medium. The red nutrient solution would gradually turn yellow because of the proliferation of Mo. Biochemical tests were performed subsequently to further verify the existence of Mo, which included glucose fermentation, digitonin test, arginine hydrolysis, tetrazolium chloride reduction test, hemadsorption test and hemolysis test.

For collecting lung tissue samples at 4th dpi and 14th respectively, all the experimental lambs were euthanized by bloodletting, after being anaesthetized by intravenous injection of Thiophene sodium following recommended dosage of 20 mg/kg. After which, their thoracic cavities were opened to expose the lungs so that gross lesions of lungs visible to the naked eye can be clearly observed and photographed. In addition, lung tissue samples collected were immediately put into liquid nitrogen tank after marking and then preserved in refrigerator (− 80 °C) until use.

### RNA extraction, library preparation, and sequencing

Commercial mir Vana™ mRNA Isolation Kit (Ambion-1561, Foster City, USA) were used to extract total RNA specimens from lung tissue under the guidance and requirements of the manufacturer’s instructions. After which, the Agencourt AMPure XP (Beckman Coulter Shanghai, China) were used to purify total RNA samples, and the purity of which were tested by 1% agarose gel electrophoresis (AGE). NanoDrop 2000 (Thermo Fisher Scientific, Beijing, China) and Agilent Bioanalyzer 2100 (Agilent Technologies, Shanghai, China) were chosen to detect RNA concentration and integrity respectively, which were used to evaluate the quality of these samples. Four micrograms of high-quality RNA were selected to synthesize cDNA, and TruSeq Stranded mRNA LT Sample Prep Kit (Illumina, Beijing, China) was used to construct libraries based on the newly synthesized cDNA.

The concentrations of individual RNA-seq libraries were verified and then pooled according to their respective index barcodes. Pooled RNA-seq libraries were sequenced as paired-end reads at 125 bp/150 bp using an Illumina HiSeq 2500 Sequencer (Illumina, Beijing, China). At the end of this experiment, we have deposited the metadata and raw sequence files in the European Nucleotide Archive (www.ebi.ac.uk/arrayexpress/), and the accession number of it was E-MTAB-7936.

### Mapping the sequence reads

NGS QC Toolkit was used to check raw sequencing reads (raw reads) we got for the first time. Low-quality reads containing poly-N, adapter sequences and low-quality sequences were discarded. The resultant quality reads after cleansing were used in all subsequent analyses. In addition, the relevant reference genome and gene model annotation files were available from NCBI Web SiteNLM, the website link is ftp://ftp.ncbi.nlm.nih.gov/genomes/all/GCF_000298735.2_Oar_v4.0/GCF_000298735.2_Oar_v4.0_genomic.fna.gz. Bowtie v2.2.3, one of the most common high-throughput short read aligner, was used to build an index for the reference genome. TopHat v2.0.12, a convenient spliced read mapper for RNA-Seq (http://tophat.cbcb.umd.edu/), was utilized to RNA-Seq reads to the above reference genome.

### Differential gene expression and functional analysis

Python HTSeq package v0.6.1 was used to determinate the number of useable RNA-Seq reads mapped to each transcript, which was one basic step for further identification of DEGs. The transcripts were assembled and their abundances were estimated based on FPKM (fragments per kilobase of exon per million fragments mapped) by using Cufflinks, an effective tool for transcriptome assembly and analysis on differential expression for RNA-Seq. Meanwhile, both the estimation of variance-mean dependence in count data and the test for differential expression were accomplished by utilizing DESeq R package (1.20.0), and the latter was based on the negative binomial distribution. EstimateSizeFactors function were used to normalize the data; NbinomTest function were used to calculate *p*-values and fold-change values after that. Meanwhile, a combined criterion widely used in bioinformatics analysis: fold changes of > 2 or < 0.667 and p-values < 0.05 was adopted in the process of statistically significant DEGs identification. What’s more, hierarchical cluster analysis on gene expression mode was conducted to screen out the specific expression patterns of DEGs related to Mo infection which was the change law of DEGs’ expression level in other words. In addition, GOseq R package was applied to explore the relationship between DEGs and changes in gene function by Gene Ontology (GO) assignments of DEGs.

### Validation by real-time reverse transcription polymerase chain reaction (qRT-PCR)

qRT-PCR was performed to determine the relative expression of 10 DEGs (TBX21, NFKB1, GATA3, BPIFA1, TREM2, IL17RB, SBD2, P2RX7, IL1RAP, PTGER2), as previously described [29]. Glyceraldehyde-3-phosphatedehydrogenase (GAPDH) was used as an endogenous reference gene for all of the above reactions, due to its stable expression in lungs of these experimental lambs. Mic Real-Time PCR Cycler (StepOnePlusTM, Thermo Fisher Scientific, Beijing, China) were employed for qRT-PCR reactions and all of them were performed in triplicate. Beacon Designer 7 (http://www.premierbiosoft.com/index.html) was used for designing the real-time PCR primers, one of the essential ingredients for reaction system (Table 3). The calculation of data obtained by normalizing the expression of target genes to the reference gene were accomplished by the comparative 2^-ΔΔCT^ method widely used with many advantages. GraphPad Prism 7, a kind of high-efficiency software for statistical analysis and mapping, was chosen to analyze (Mann–Whitney U test) the data gained after qRT-PCR. And *p*-value less than 0.05 indicated that differences were significant statistically.

**Table 3:** Details of the RT-qPCR assays used to validate the RNA-Seq results

Accession number^a^	Gene symbol	Gene name	Sequence (5′➔ 3′)
XM_004018378	IL17RB	Interleukin17 receptor B	F:TTCTCAACTACCACACTA R:AAGGATAACTTCACTTCTG
XM_012097111.2	IL1RAP	interleukin 1 receptor accessory protein	F:CCAAGTGTATGAAGATGAA R:TACCAGATGAGAGTAAGG
XM_015101624.1	P2RX7	purinergic receptor P2X7	F:AGAGTCAAGAGATTCAGT R:GTAGGTTGGCTAATAAGG
XM_004018807.3	TREM2	triggering receptor expressed on myeloid cells 2	F:AATACCGATGAGGAGAAG R:CTGTGGAAATGGAAGAAC
NM_001198545.1	SBD2	beta defensin 2	F:TGGGTCAGGATTTACTCAT R:CTTGGTCAGCACACAGAT
NM_001278560.1	PTGER2	prostaglandin E receptor 2	F:GTGTCTTGTGATTGCTTAT R:CCAACTGAACCTACTCTT
NM_001301405.2	BPIFA1	BPI fold containing family A member 1	F:TCTCTGCTTGATGGATTG R:CTCAGGAAGGACATTATTCA
NM_001252183.1	GATA3	GATA binding protein 3	F:CGTGGTGTCTGTGTTCTC R:ATAGGGAATGTGAGTCTGAATG
XM_012179609.2	NFKB1	nuclear factor kappa B subunit 1	F:CTAAGATATTCCGAGCAGAA R:CTAACCTACAGTGTCCTATG
XM_004012818.3	TBX21	T-box 21	F:CAGAACGCCGAGATTACT R:ACGGATACATACATGGATTCA
NM_001190390.1	GAPDH	glyceraldehyde-3-phosphate dehydrogenase	F:TGCCAAGTATGATGAGAT R:TCAGTGTAGCCTAGAATG

^a^Most relevant information in the table referred to Entrez Gene [http://www.ncbi.nlm.nih.gov/sites/entrez?db=gene], NCBI’s repository for gene-specific information

## Supplementary Information

**Additional file 1: Table S1.** The top upregulated genes (Sample_4d_Z-vs-Sample_4d_B). **Table S2.** The top downregulated genes (Sample_4d_Z-vs-Sample_4d_B). **Table S3.** The top upregulated genes (Sample_Z_14d-vs-Sample_B_14d). **Table S4.** The top downregulated genes (Sample_Z_14d-vs-Sample_B_14d).

## Data Availability

All the sequencing data have been uploaded to the European Nucleotide Archive (ENA) website (www.ebi.ac.uk/arrayexpress/), and the accession number is E-MTAB-7936.

## Abbreviations

Bbs: Bashbay sheep
Ahs: Argali hybrid sheep
Mo: *Mycoplasma ovipneumoniae*
dpi: Day post infection
CCU: Color change unit
DEGs: Differentially expressed genes
GO: Gene Ontology
SPLUNC1: Short palate, lung and nasal epithelium clone 1
