# Supplementary material for: Comparative analysis on lung transcriptome of Mycoplasma ovipneumoniae (Mo) - infected Bashbay sheep and argali hybrid sheep
Source: BMC Vet Res. 2021 Oct 13;17:327. doi: 10.1186/s12917-021-03040-3 (PMC8511284; doi:10.1186/s12917-021-03040-3)
Supplement: Supplementary file 1 — Additional file 1: Table S1. The top upregulated genes (Sample_4d_Z-vs-Sample_4d_B). Table S2. The top downregulated genes (Sample_4d_Z-vs-Sample_4d_B). Table S3. The top upregulated genes (Sample_Z_14d-vs-Sample_B_14d). Table S4. The top downregulated genes (Sample_Z_14d-vs-Sample_B_14d). [file 12917_2021_3040_MOESM1_ESM.pdf]

Supplementary materials

Table S1. The top upregulated genes (Sample_4d_Z-vs-Sample_4d_B)

id	term	category	pval	Enrichment_score	Gene
GO:0006812	cation transport	biological_process	0.000561	7.856739	ATP7A; P2RX7; SLC30A7
GO:0006486	protein glycosylation	biological_process	0.000652	7.554557	B3GALT1; B4GALNT2; FUT11
GO:0000122	negative regulation of transcription from RNA polymerase II promoter	biological_process	0.015379	2.304029	ALX1; BEND3; ESR2; LOC101104566; PRDM5; TRIM29
GO:0045893	positive regulation of transcription, DNA-templated	biological_process	0.026778	2.189727	ALX1; ARNTL2; ESR2; NR1D2; TP53INP1
GO:0006955	immune response	biological_process	0.027169	2.63649	C6; DQA; ENPP3
GO:0006351	transcription, DNA-templated	biological_process	0.028365	2.338315	ARNTL2; ESR2; FOXN2; NR1D2
GO:0007155	cell adhesion	biological_process	0.041074	2.310806	EFNB2; ITGA4; LOC101102480
GO:0010628	positive regulation of gene expression	biological_process	0.044106	2.257684	LOC101110922; P2RX7; SLC6A4
GO:0008152	metabolic process	biological_process	0.063065	1.520855	AFMID; ARSJ; BCHE; ENPP3; GFPT1; HS3ST3A1; LOC101108953; PDE1C; PDE5A; TMPPE
GO:0008284	positive regulation of cell proliferation	biological_process	0.072759	1.906976	DDR2; EIF5A2; TET1
GO:0005887	integral	cellular_component	0.007002	2.949226	CLDN1; DDR2;

	component of plasma membrane				HTR1B; P2RX7; SLC6A4
GO:0005794	Golgi apparatus	cellular_component	0.054074	1.761601	ALX1; ATP7A; ATP8B1; B3GALT1; FUT11; SLC30A7
GO:0005925	focal adhesion	cellular_component	0.074804	1.888639	DDR2; EFNB2; ITGA4
GO:0048471	perinuclear region of cytoplasm	cellular_component	0.093346	1.745942	ATP7A; ENPP3; SLC30A7
GO:0005813	centrosome	cellular_component	0.157274	1.433712	CEP135; ERCC6L; LOC101108953
GO:0016021	integral component of membrane	cellular_component	0.187411	1.166417	ATP7A; ATP8B1; B3GALT1; CLDN1; DQA; FUT11; GABRA1; HS2ST1; HTR1B; ITGA4; LOC101102480; LOC101111847; MS4A13; ND4; P2RX7; PTGER2; SLC30A7; SLC6A4; SLCO1A2
GO:0005829	cytosol	cellular_component	0.198988	1.27379	AFMID; LOC101108953; RASSF9; SLC6A4; TP53INP1
GO:0005886	plasma membrane	cellular_component	0.274461	1.116807	ATP7A; ATP8B1; CLDN1; EFNB2; ERCC6L; GABRA1;

					HTR1B; ITGA4; LOC101102480; P2RX7; PAG1; SLC6A4
GO:0016020	membrane	cellular_component	0.468494	0.988269	ATP7A; ATP8B1; B3GALT1; CLDN1; DDR2; DQA; EFNB2; EML4; ERCC6L; FUT11; GABRA1; HTR1B; IL1RAP; ITGA4; LARP4; LOC101102480; LOC101108953; LOC101111847; ND4; P2RX7; SLC6A4; SLCO1A2
GO:0070062	extracellular exosome	cellular_component	0.661253	0.837784	C6; DDR2; ENPP3; FUT11; GFPT1; ITGA4; NHLRC3; RASSF9; SLC30A7; TAB3
GO:0004114	3',5'-cyclic-nucleotide phosphodiesterase activity	molecular_function	8.98E-06	21.82428	LOC101108953; PDE1C; PDE5A
GO:0008081	phosphoric diester hydrolase activity	molecular_function	0.00026	9.581389	LOC101108953; PDE1C; PDE5A
GO:0016787	hydrolase activity	molecular_function	0.001085	2.459073	AFMID; ATP7A; ATP8B1; BCHE; ENPP3; ERCC6L; LOC101108953;

					PDE1C; PDE5A; TLL1; TMPPE; USP45
GO:0016757	transferase activity, transferring glycosyl groups	molecular_function	0.005336	4.269967	B3GALT1; B4GALNT2; FUT11
GO:0043565	sequence-specific DNA binding	molecular_function	0.013246	2.373637	ALX1; ESR2; FOXN2; GATAD2B; NR1D2; PRDM5
GO:0003700	transcription factor activity, sequence-specific DNA binding	molecular_function	0.017254	2.251215	ALX1; ARNTL2; ESR2; FOXN2; GATAD2B; NR1D2
GO:0008270	zinc ion binding	molecular_function	0.028731	1.732085	ESR2; FBXO30; GATAD2B; NR1D2; PEG10; TAB3; TET1; TLL1; TRIM29; USP45
GO:0046872	metal ion binding	molecular_function	0.035431	1.540537	ATP7A; ATP8B1; ENPP3; ESR2; FBXO30; LOC101108953; LOC101110922; NR1D2; PDE1C; PDE5A; PRDM5; TLL1; USP45; ZNF654
GO:0005509	calcium ion binding	molecular_function	0.066291	1.75061	LOC101102480; MICU3; MMRN1; SMOC1; TLL1
GO:0005515	protein binding	molecular_function	0.103728	1.103581	ALX1; ANKRD44; ARHGAP42;

	ARNTL2; ATP7A; ATP8B1; BEND3; C6; CEP135; CLDN1; DDR2; DENND5B; DOK6; DQA; EFNB2; EIF5A2; EML4; EPM2AIP1; ERCC6L; ESR2; FBXO30; FOXN2; FUT11; GABRA1; GATAD2B; GTF3C4; HTR1B; IL1RAP; ITGA4; KPNA5; LARP4; LCA5; LOC101104566; LOC101108953; LRRC8B; LRRN1; MMRN1; NHLRC3; NR1D2; P2RX7; PAG1; PAIP2B; PDE5A; PLEKHS1; PRDM5; RASSF9; RCAN3; SEL1L; SGTB; SLC6A4; SLCO1A2; SLITRK1; SMOC1; STK31; TAB3;

					TLL1; TP53INP1; TRIM29

Table S2. The top downregulated genes (Sample_4d_Z-vs-Sample_4d_B)

id	term	category	pval	Enrichment_score	Gene
GO:0006810	transport	biological_process	0.033085	2.210357	ALB; FABP4; LCN2; LOC101112166
GO:0070062	extracellular exosome	cellular_component	0.000863	2.371573	ALB; ATP6V0D2; CBLC; CTSL; FABP4; KLK12; KRT78; KRT7; LYVE1; SERPINA1; TFF2; TGM1
GO:0005576	extracellular region	cellular_component	0.001956	3.739678	ALB; LCN2; PLA2G10; PTHLH; SBD2
GO:0005615	extracellular space	cellular_component	0.007689	2.828731	ALB; CTSL; KRT78; LOC101106229; SERPINA1
GO:0005794	Golgi apparatus	cellular_component	0.013878	2.770381	ALB; GPR143; PTHLH; SERPINA1
GO:0005886	plasma membrane	cellular_component	0.683314	0.65863	FCAR; GPR143; LYVE1
GO:0005737	cytoplasm	cellular_component	0.706979	0.755866	ALB; FABP4; HSPA6; KRT7; PTHLH; SOD1
GO:0005634	nucleus	cellular_component	0.720345	0.74643	ALB; ARMC12; CBLC; GCM1; KRT7; SOD1
GO:0016021	integral component of membrane	cellular_component	0.932883	0.434455	GPR143; LOC101112166; TREM2

GO:0016020	membrane	cellular_component	0.992228	0.317905	ATP6V0D2; GPR143; LOC105608615
GO:0005215	transporter activity	molecular_function	0.000842	7.020396	FABP4; LCN2; LOC101112166
GO:0005509	calcium ion binding	molecular_function	0.031981	2.477787	CBLC; NELL2; PLA2G10
GO:0016787	hydrolase activity	molecular_function	0.053682	1.93363	CTSL; KLK12; LOC101112822; PLA2G10
GO:0046872	metal ion binding	molecular_function	0.340556	1.038311	CBLC; CYP3A24; SOD1; TGM1
GO:0005515	protein binding	molecular_function	0.47461	0.987466	ALB; ASGR2; ATP6V0D2; C4H7orf31; CBLC; CHAD; CTSL; FCRL6; GCM1; GPR143; HSPA6; KRT7; LOC101103260; LOC101116157; LOC101117062; LRP11; LYVE1; NELL2; SERPINA1; TFF2; TGM1; TREM2

Table S3. The top upregulated genes (Sample_Z_14d-vs-Sample_B_14d)

id	term	category	pval	Enrichment_score	Gene
GO:0008217	regulation of blood pressure	biological_process	2.31E-05	17.45942	ACTA2; NPY1R; POMC
GO:0055114	oxidation-reduction process	biological_process	0.00174	2.707191	COX1; CYP17A1; CYP2A6; DCXR; HSD17B3; LOC101102548; LOC101104591; LOC101109936; ND6

GO:0010628	positive regulation of gene expression	biological_process	0.041222	2.307854	ACTA2; LOC101103781; PLP1
GO:0008152	metabolic process	biological_process	0.055408	1.554652	AFMID; ARSE; ATP13A5; DCXR; GCNT7; HSD17B3; LOC101108949; PYGM; SLC27A2; SLC27A6
GO:0043547	positive regulation of GTPase activity	biological_process	0.06923	1.939936	ARHGAP22; RGL3; RGS18
GO:0006508	proteolysis	biological_process	0.21038	1.262788	LOC101105864; MCP-3; MMP23B; TMPRSS4
GO:0006355	regulation of transcription, DNA-templated	biological_process	0.332374	1.060662	FOXJ1; FOXN4; MYCL; SOX2; VGLL1
GO:0007165	signal transduction	biological_process	0.461157	0.956111	ARHGAP22; GRB14; HPCAL4; MRGPRF; NPY1R; POMC; RGL3; SCTR
GO:0045944	positive regulation of transcription from RNA polymerase II promoter	biological_process	0.538996	0.781258	FOXJ1; POMC; SOX2
GO:0007186	G-protein coupled receptor signaling pathway	biological_process	0.741083	0.653751	MRGPRF; NPY1R; RGS18; SCTR
GO:0043234	protein complex	cellular_component	0.052969	2.124691	ACTA2; CLSTN3; NEK2

GO:0005813	centrosome	cellular_component	0.054183	1.954096	CCDC13; HEPACAM2; LOC101103781; NEK2
GO:0005925	focal adhesion	cellular_component	0.070201	1.930609	ARHGAP22; CNN1; FLNC
GO:0005739	mitochondrion	cellular_component	0.109897	1.394329	C5H5orf63; COX1; COX3; ENDOG; LOC101108949; MINOS1; ND6; SLC22A4; SLC27A2
GO:0005783	endoplasmic reticulum	cellular_component	0.163137	1.38352	LOC101109936; SLC27A2; THBS4; TMEM119
GO:0005615	extracellular space	cellular_component	0.223282	1.225783	ACTA2; CTHRC1; POMC; THBS4; TNFSF13
GO:0016021	integral component of membrane	cellular_component	0.323713	1.066828	ATP13A5; CNTNAP1; COX1; COX3; FER1L6; GAL3ST4; JAML; LOC101118175; MRGPRF; ND6; NPY1R; PLP1; SCTR; SLC22A4; SLC27A2; SLC27A6; SLC38A11
GO:0005576	extracellular region	cellular_component	0.372254	0.972316	PLA2G2D; POMC; THBS4
GO:0070062	extracellular exosome	cellular_component	0.506751	0.942042	ACTA2; BCAS1; C24H16orf89; CLSTN3; FCER2;

					GAL3ST4; MRGPRF; PYGM; SLC27A2; THBS4; TNFSF13
GO:0005829	cytosol	cellular_component	0.538996	0.781258	AFMID; C24H16orf89; SOX2
GO:0004497	monooxygenase activity	molecular_function	8.90E-05	8.777413	CYP17A1; CYP2A6; LOC101104591; LOC101109936
GO:0016491	oxidoreductase activity	molecular_function	0.000174	3.639577	COX1; CYP17A1; CYP2A6; DCXR; HSD17B3; LOC101102548; LOC101104591; LOC101109936; ND6
GO:0050660	flavin adenine dinucleotide binding	molecular_function	0.000315	9.126515	LOC101102548; LOC101104591; LOC101109936
GO:0005506	iron ion binding	molecular_function	0.001267	4.957613	COX1; CYP17A1; CYP2A6; LOC101102548
GO:0003824	catalytic activity	molecular_function	0.002361	2.76704	ARSE; CKB; DCXR; LOC101102548; PYGM; RIMKLA; SLC27A2; SLC27A6
GO:0020037	heme binding	molecular_function	0.003721	4.724314	COX1; CYP17A1; CYP2A6
GO:0005102	receptor binding	molecular_function	0.009719	3.077139	NXPH3; POMC; SLC27A2;

					TNFSF9
GO:0016787	hydrolase activity	molecular_function	0.048742	1.675813	AFMID; ATP13A5; ENDOG; LOC101105864; MCP-3; PLA2G2D; PTPN20; TMPRSS4
GO:0008233	peptidase activity	molecular_function	0.102528	1.687255	LOC101105864; MCP-3; TMPRSS4
GO:0003700	transcription factor activity, sequence-specific DNA binding	molecular_function	0.119923	1.534161	FOXJ1; FOXN4; MYCL; SOX2

Table S4. The top downregulated genes (Sample_Z_14d-vs-Sample_B_14d)

id	term	category	pval	Enrichment_score	Gene
GO:0046777	protein autophosphorylation	biological_process	0.005011	4.34596	DDR2; KIT; TRPM7
GO:0006468	protein phosphorylation	biological_process	0.008004	2.430791	CSNK1G1; DDR2; KIT; MAML1; PDK4; RASSF2; TRPM7
GO:0007264	small GTPase mediated signal transduction	biological_process	0.030891	2.530882	ARHGAP35; ARL5A; GEM
GO:0008284	positive regulation of cell proliferation	biological_process	0.055685	2.088592	DDR2; EIF5A2; KIT
GO:0006886	intracellular protein transport	biological_process	0.058951	2.04881	ARL5A; CHML; FAM135A

GO:0055085	transmembrane transport	biological_process	0.097319	1.639048	ATP6V1G3; SLC25A44; TRPC5; TRPM7
GO:0045892	negative regulation of transcription, DNA-templated	biological_process	0.100252	1.700593	ARHGAP35; LOC101117035; NR1D2
GO:0045944	positive regulation of transcription from RNA polymerase II promoter	biological_process	0.1487	1.395104	DLX3; GABPB2; LOC101117035; MAML1; TAF2
GO:0000122	negative regulation of transcription from RNA polymerase II promoter	biological_process	0.214261	1.26173	ARHGAP35; LOC101117035; TAF9B
GO:0008152	metabolic process	biological_process	0.250724	1.165989	BCAT1; EOGT; GLA; LOC101115808; PDHA1; SPTLC3; USF3
GO:0005669	transcription factor TFIID complex	cellular_component	2.09E-06	30.73214	TAF2; TAF4B; TAF9B
GO:0005576	extracellular region	cellular_component	0.068165	1.736279	C1QTNF3; GLA; LOC101105809; SBD2; TIMP3
GO:0043231	intracellular membrane-bounded organelle	cellular_component	0.086959	1.697239	EIF5A2; FOXN2; MAML1; PDHA1
GO:0005794	Golgi apparatus	cellular_component	0.199976	1.286248	ATP7A; FAM198B; GLA; LOC101117035
GO:0005886	plasma membrane	cellular_component	0.272744	1.121239	ATP6V1G3; ATP7A; CACNB4; DENND4C; EFNB2; KLF9; LYVE1;

					PARD6B; RGS7BP; TRPM7; UBQLN1
GO:0005730	nucleolus	cellular_component	0.344336	1.012353	LARP4B; N4BP1; TAF4B
GO:0005654	nucleoplasm	cellular_component	0.362982	1.039251	CHML; KLF9; LOC101117035; MAML1; TAB3; TAF4B; UBN2; UBQLN1
GO:0005829	cytosol	cellular_component	0.484494	0.837062	ATP6V1G3; DENND4C; LARP4B
GO:0005622	intracellular	cellular_component	0.541427	0.849625	ARHGAP35; ARL5A; GEM; LOC101114805; ZC3H11A
GO:0005634	nucleus	cellular_component	0.577168	0.924151	DLX3; EPM2AIP1; FOXN2; GABPB2; GEM; KLF9; LOC101117035; MAML1; N4BP1; NR1D2; PARD6B; RASSF2; RGS7BP; TIMP3; UBN2; ZBTB6
GO:0003676	nucleic acid binding	molecular_function	0.007956	2.087579	KIAA0430; KLF9; LARP4B; LARP4; LOC101108538; LOC101114805; LOC101117035; MYEF2;

					RBMS3; ZBTB6
GO:0004672	protein kinase activity	molecular_function	0.04622	1.917335	CSNK1G1; DDR2; KIT; PDK4; RASSF2
GO:0005525	GTP binding	molecular_function	0.087621	1.78527	ARHGAP35; ARL5A; GEM
GO:0003700	transcription factor activity, sequence-specific DNA binding	molecular_function	0.096432	1.643744	FOXN2; LOC101117035; NR1D2; TAF4B
GO:0000166	nucleotide binding	molecular_function	0.096872	1.404669	ARL5A; ATP7A; CSNK1G1; GEM; KIAA0430; KIT; LOC101108538; LOC101115808; MYEF2; RBMS3
GO:0046982	protein heterodimerization activity	molecular_function	0.105753	1.667636	GABPB2; TAF4B; TAF9B
GO:0005515	protein binding	molecular_function	0.110643	1.104488	ARAP2; ARHGAP42; ATP7A; C1QTNF3; CACNB4; CSNK1G1; DDR2; DENND5B; DNAJC6; EFNB2; EIF5A2; EML5; EPM2AIP1; FBXO30; FOXN2; FRMD6; GABPB2; GEM; GLA;

					KIT; LARP4B; LARP4; LOC101105256; LOC101114805; LOC101117035; LRCH1; LRRC32; LYVE1; MAML1; N4BP1; NHLRC2; NHLRC3; NR1D2; PARD6B; RAD54L2; RANBP10; RASSF2; RGS7BP; RNF168; SPHKAP; TAB3; TAF2; TAF9B; TIMP3; TMTC1; TRPC5; TRPM7; TULP4; UBQLN1; WIPF2; XIAP; ZBTB6; ZC3H11A
GO:0046872	metal ion binding	molecular_function	0.122402	1.3257	ATP7A; FBXO30; KLF9; LOC101105256; LOC101114805; LOC101117035; NR1D2; RNF168; XIAP; ZBTB6; ZC3H11A
GO:0016772	transferase activity, transferring phosphorus-	molecular_function	0.13401	1.478522	CSNK1G1; DDR2; KIT;

	containing groups				TRPM7
GO:0008270	zinc ion binding	molecular_function	0.155915	1.327932	FBXO30; LOC101105256; NR1D2; RNF168; TAB3; TAF2; XIAP
